# Autologous precision-cut lung slice co-culture models for studying macrophage-driven fibrosis

**DOI:** 10.3389/fphys.2025.1526787

**Published:** 2025-01-31

**Authors:** So-Yi Chang, Wen-Hsin Chang, David C. Yang, Qi-Sheng Hong, Ssu-Wei Hsu, Reen Wu, Ching-Hsien Chen

**Affiliations:** ^1^ Division of Pulmonary, Critical Care, and Sleep Medicine, Department of Internal Medicine, University of California Davis, Davis, CA, United States; ^2^ Division of Nephrology, Department of Internal Medicine, University of California Davis, Davis, CA, United States

**Keywords:** precision-cut lung slices (PCLS), macrophage recruitment, fibrosis inducers, co-culture models, pulmonary fibrosis

## Abstract

Precision-cut lung slices (PCLS) are commonly used as an *ex vivo* model to study lung fibrosis; however, traditional models lack immune cell infiltration, including the recruitment of monocytes and macrophages, which are critical for inflammation and fibrosis. To address this limitation, we developed novel autologous PCLS-immune co-culture models that better replicate the processes of inflammation, repair, and immune cell recruitment associated with fibrosis. Fibrotic responses to nicotine, cigarette smoke extract (CSE), and a fibrosis-inducing cocktail (FC) were first evaluated in PCLS containing only tissue-resident macrophages, with upregulation of α-SMA-expressing fibroblasts confirmed by immunofluorescence and Western blotting, and collagen deposition quantified using Sirius Red staining. To study macrophage recruitment, we employed an indirect co-culture model using transwells to approximate blood vessel function. Chemotactic studies revealed increased migration of autologous bone marrow-derived macrophages (BMDMs) toward and infiltration into CSE-injured PCLS. In a direct co-culture model simulating the repair phase of fibrosis, PCLS exposed to CSE and FC showed further increased collagen deposition in the presence of autologous BMDMs, but not heterologous ones. These findings suggest that our novel PCLS-immune co-culture models provide a platform for studying macrophage involvement in fibrosis and offer potential for developing macrophage-targeted therapeutic strategies in pulmonary fibrosis.

## Introduction

Lung fibrosis, most commonly manifested as idiopathic pulmonary fibrosis (IPF), is characterized by the abnormal thickening and scarring of interstitial lung tissue, leading to impaired gas exchange. Its development is influenced by a combination of genetic predispositions and external factors, such as smoking and exposure to hazardous materials, which contribute to lung tissue injury (Martinez et al., 2017; Karampitsakos et al., 2023). The pathogenesis of lung fibrosis begins with damage to epithelial and endothelial cells, triggering a repair response that involves the recruitment of platelets and fibroblasts to the injury site. This process is followed by the infiltration of inflammatory cells, including lymphocytes and macrophages, which secrete pro-fibrotic growth factors, cytokines, and chemokines like TGF-β and IL-13. The excessive expression of these factors leads to an unregulated fibrotic response, a hallmark of the disease (Sari et al., 2022; Mutsaers et al., 2023; Kolanko et al., 2024).

Given the poor prognosis and limited treatment options for IPF, there is an urgent need to explore its pathogenesis and identify new therapeutic targets. Macrophages, innate immune cells that are essential for development, homeostasis, and immune regulation, play a critical role in pulmonary fibrosis through their interactions with cytokines and chemokines in fibrotic tissues (Heukels et al., 2019; Shenderov et al., 2021; Mutsaers et al., 2023). Tissue-resident macrophages act as first responders, initiating inflammatory responses, while recruited monocytes differentiate into macrophages at sites of injury. These macrophages collaborate with fibroblasts, secreting pro-fibrotic cytokines such as TGF-β, which drive fibroblast activation and tissue remodeling (Wynn and Vannella, 2016; Gu et al., 2022; Sari et al., 2022). Numerous studies have underscored the importance of macrophage regulation in fibrotic progression (Byrne et al., 2016; Isshiki et al., 2023; Perrot et al., 2023), highlighting the need for further research into the mechanisms of macrophage activation and their interactions with environmental stressors.

Although bleomycin is widely used to induce pulmonary fibrosis in mouse models (Moeller et al., 2008), triggering an inflammatory response that escalates fibroblast activation and subsequent fibrosis (Kolb et al., 2020), this *in vivo* approach does not fully capture the progression of fibrosis or elucidate specific underlying mechanisms (Ayilya et al., 2023). Precision-cut lung slices (PCLS), derived from human or mouse lung tissue, offer a valuable *ex vivo* platform for studying lung fibrosis by providing a three-dimensional (3D) representation of the cellular and structural organization of lung tissue (Liu et al., 2019; Alsafadi et al., 2020; Liu et al., 2021; Liu et al., 2022; Lehmann et al., 2024). In this technique, lung tissue, either whole or individual lobes, is embedded in agar, thinly sliced, and maintained in medium, allowing for the direct observation of resident lung and immune cells in their natural environment (Alsafadi et al., 2017; Gerckens et al., 2021). Despite these advantages, PCLS face limitations, such as the absence of blood and lymphatic circulation, which restricts the recruitment of immune cells in response to injury. These constraints in both *in vivo* and *ex vivo* models underscore the need for more representative models to comprehensively investigate the mechanisms underlying fibrosis.

Utilizing an autologous PCLS-immune co-culture model, our study aimed to investigate lung fibrosis and identify key cellular contributors, such as macrophages, within lung tissue following exposure to fibrosis inducers. We addressed the limitations of traditional PCLS models, particularly the lack of monocyte and macrophage recruitment to injured lung tissue. To overcome these challenges, we developed two co-culture models designed to replicate the stages of inflammation, repair, and immune cell recruitment: a direct co-culture that simulates the repair phase of fibrosis and an indirect co-culture that mimics blood vessel function, allowing for the observation of immune cell recruitment to injury sites. By addressing the limitations of existing PCLS models, this study presents a novel and effective approach for exploring the mechanisms underlying pulmonary fibrosis.

## Materials and methods

### Reagents and antibodies

All reagents and antibodies used in this study are described in the Supplementary Materials and Methods in the online Supplementary Material.

### Mouse precision-cut lung slices (PCLS)

The procedures of all mouse experiments were approved by the Institutional Animal Care and Use Committee (IACUC) of UC Davis. Euthanasia was performed on 8-week-old male C57BL/6J mice (Jackson Laboratory, Sacramento, CA, United States) via intraperitoneal injection of a lethal dose of sodium pentobarbital (>100 mg/kg). Subsequently, the lungs were perfused with 2–3 mL of 1.5% low-melting-point agarose dissolved in sterile PBS through the trachea. Once the agarose had solidified, the lung lobes were excised and immersed in sterile, ice-cold PBS. These lobes were then embedded in 2% agarose and sectioned using a vibratome (VF-300; Precisionary Instruments, Greenville, NC, United States). Slices 300 μm thick were prepared for immunofluorescence staining, Western blotting, Sirius Red/Fast Green staining extraction, and co-culture assays, while 150 μm slices were used for histological imaging of Sirius Red/Fast Green staining. Sectioning was performed with the interior part of the lung, connected to the trachea, attached to the plunger to optimize visualization of large airways and parenchyma (Lyons-Cohen et al., 2017). Typically, 15 to 20 lung slices, each 300 μm thick, were obtained from a single lobe. The slices were transferred to pre-warmed PCLS medium (comprising DMEM/F12 supplemented with 1X ITS-G, 0.1% penicillin/streptomycin) and incubated overnight. The next day, the slices were moved to fresh medium to eliminate dead cells, damage signals, and inflammatory cytokines, and were subsequently used for further experimentation. All lung slices used in each experiment were derived from the same mouse, with each treatment group comprising three to eight lung slices. All assays were performed using PCLS obtained from three different mice. A total of 21 mice were utilized for the formal experiments.

### PCLS viability assay

Well-prepared PCLS were cultured in medium for 2 weeks, with the medium replaced every 3 days. On day 14 post-slicing, PCLS viability was assessed using the LIVE/DEAD™ Viability/Cytotoxicity Kit for mammalian cells (Thermo Fisher Scientific, Waltham, MA, United States). Briefly, 5 µL of Calcein AM ester and 20 µL of ethidium homodimer-1 were added to 10 mL of PBS to prepare the staining solution. After removing the culture medium, PCLS were incubated with 500 µL of the staining solution for 30 min at 20°C–25°C. Fluorescent imaging was performed using a microscope (Zeiss, Observer. A1). Green fluorescence indicated live cells, while red fluorescence indicated dead cells.

### Exposure of cultured PCLS to mimic lung injury and fibrotic environment

PCLS were exposed to nicotine, cigarette smoke extract (CSE), and a fibrosis-inducing cocktail (FC) following previously described protocols (Alsafadi et al., 2017; Gerckens et al., 2021). In brief, research cigarettes (Kentucky Tobacco R&D Center, Lexington, KY, United States) were lit, and mainstream smoke was drawn into a 60 mL catheter-tip syringe containing 5 mL of medium. This medium was vigorously shaken for 20 s, and the procedure was repeated four times. The resulting medium, filtered through a 0.22 μm filter, was designated as 100% CSE. The FC was composed of 5 ng/mL TGF-β, 10 ng/mL TNF-α, 5 μM PDGF-BB, and 5 μM LPA. Prepared PCLS were exposed to 10 μM nicotine (Dasgupta et al., 2009), 10% CSE (Kanaji et al., 2014), or FC (Alsafadi et al., 2017) for 3 days, followed by a medium change and an additional 3-day exposure, after which the tissue was harvested.

### Isolation of mouse bone marrow-derived macrophages (BMDMs)

Femurs and tibiae from 8-week-old male mice were used to harvest bone marrow by flushing with RPMI medium using a 25-gauge syringe. The detailed experimental procedure for isolating mouse BMDMs is provided in the Supplementary Materials and Methods.

### *Ex vivo* co-culture system of bone marrow-derived macrophages and PCLS

This *ex vivo* model, designed to investigate acute inflammation recruitment, utilized a transwell system (5 µm pore size, Costar, Cambridge, MA, United States). The transwell membrane thickness is proprietary to the manufacturer. A 24-hour incubation period was chosen to allow macrophage activation and migration while minimizing proliferation, based on macrophage doubling times of 20–30 h (Chitu et al., 2011) and chemotaxis studies supporting migration within 2–24 h (Arts and Netea, 2016). Injured PCLS in the bottom chamber mimic a distal injured lung. Detailed co-culture procedures are provided in the Supplementary Materials and Methods.

### Sirius red/fast green collagen staining

For collagen detection in PCLS, we utilized a modified protocol from the Sirius Red/Fast Green collagen staining kit (Chondrex, WA, United States). The detailed experimental procedure for this assay is provided in the Supplementary Materials and Methods. Histological quantification was conducted in multiple random areas per section and reviewed by an independent individual blinded to the sample identities.

### Immunofluorescence and immunoblotting

PCLS treated with nicotine, CSE, and FC for 6 days were washed with PBS and then subjected to either immunofluorescence or immunoblotting. The detailed experimental procedures for both assays are provided in the Supplementary Materials and Methods.

### Statistical analysis

Data from at least three independent experiments are presented as the mean ± standard deviation (SD). Quantitative variables were analyzed using an unpaired two-tailed Student’s t-test. All analyses were performed using GraphPad Prism software (version 8.0.2, Boston, MA, United States). Statistical tests were two-sided, and *p*-values <0.05 were considered statistically significant.

## Results

### Myofibroblast activation in precision-cut lung slices exposed to fibrosis-inducing agents

**FIGURE 1:**
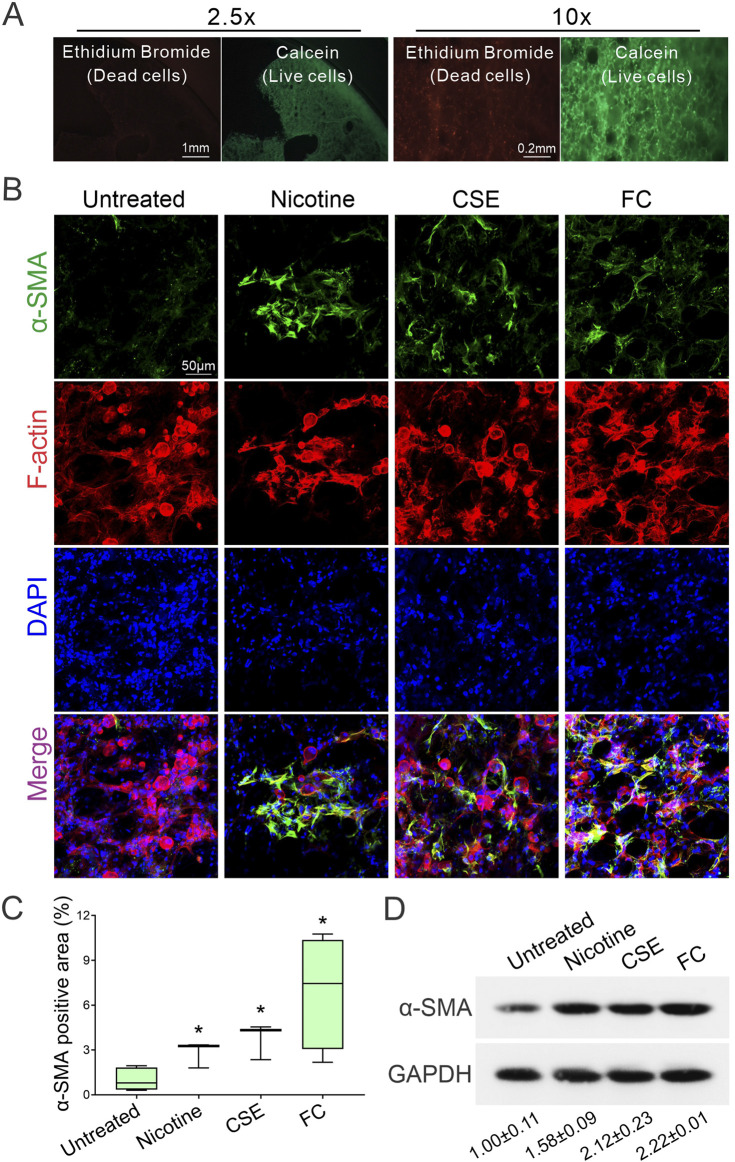
Differential induction of myofibroblast activation in precision-cut lung slices (PCLS) by fibrotic reagents. **(A)** Representative images show PCLS viability on day 14 post-slicing. Green fluorescence from Calcein marks live cells, while red fluorescence from ethidium bromide indicates dead cells. **(B)** Immunofluorescence staining was used to visualize α-SMA (green). Actin filaments were stained with phalloidin (red), and nuclei were counterstained with DAPI (blue). Images were acquired using confocal microscopy at ×40 magnification. **(C)** Quantification of α-SMA fluorescence intensity was conducted on confocal microscopy images to compare myofibroblast activation among the treatment groups (mean ± SD; n = 3; *p < 0.05). **(D)** Western blot analysis shows increased α-SMA protein levels across groups treated with fibrotic reagents. The intensity of α-SMA protein signals was quantified using ImageJ software and normalized to the internal control, GAPDH.

The utility of precision-cut lung slices (PCLS) as an *ex vivo* model is supported by their viability, which is maintained for at least 14 days (Figure 1A). To investigate fibrotic responses in PCLS, we evaluated the effects of various fibrosis inducers on myofibroblast activation. Mouse PCLS were exposed to either 10 µM nicotine, 10% cigarette smoke extract (CSE), or a fibrosis-inducing cocktail (FC) containing TGF-β, PDGF-BB, TNF-α, and LPA, which are known mediators of lung fibrosis (Jensen et al., 2012; Alsafadi et al., 2017; Song et al., 2019). After 6 days of exposure, actin filaments were stained with phalloidin to delineate cell boundaries and highlight fibrotic lesions, while nuclei were counterstained with DAPI to visualize total cell numbers. Myofibroblast activity was assessed by immunofluorescence staining for alpha smooth muscle actin (α-SMA), a marker of myofibroblast presence. The untreated control showed baseline α-SMA expression, whereas nicotine-treated PCLS exhibited increased α-SMA levels, indicating myofibroblast activation. This response was further elevated in the CSE group and was most pronounced in the FC group, which showed a marked increase in α-SMA expression (Figure 1B). The co-localization of F-actin and α-SMA signals in the stimulated groups underscores the association between cytoskeletal remodeling and myofibroblast activation in response to fibrotic stimuli. Quantification of α-SMA fluorescence intensity using confocal microscopy confirmed significant myofibroblast activation across the treatment groups (Figure 1C). Nicotine-treated PCLS demonstrated a notable rise in α-SMA signal compared to the untreated control, indicating fibrotic onset. The CSE group showed a greater increase, while the FC group exhibited the highest α-SMA intensity, significantly exceeding both untreated control and nicotine-treated samples. Western blot analysis further validated these findings, showing increased α-SMA protein levels across the different treatments (Figure 1D). These results demonstrate that exposure to nicotine, CSE, and FC induces differential fibrotic responses in PCLS, as indicated by increased α-SMA expression. This pattern of myofibroblast activation underscores the utility of the PCLS model in simulating lung fibrosis, supporting its application in fibrosis research and therapeutic target discovery.

### Collagen deposition in precision-cut lung slices following exposure to fibrosis inducers

**FIGURE 2:**
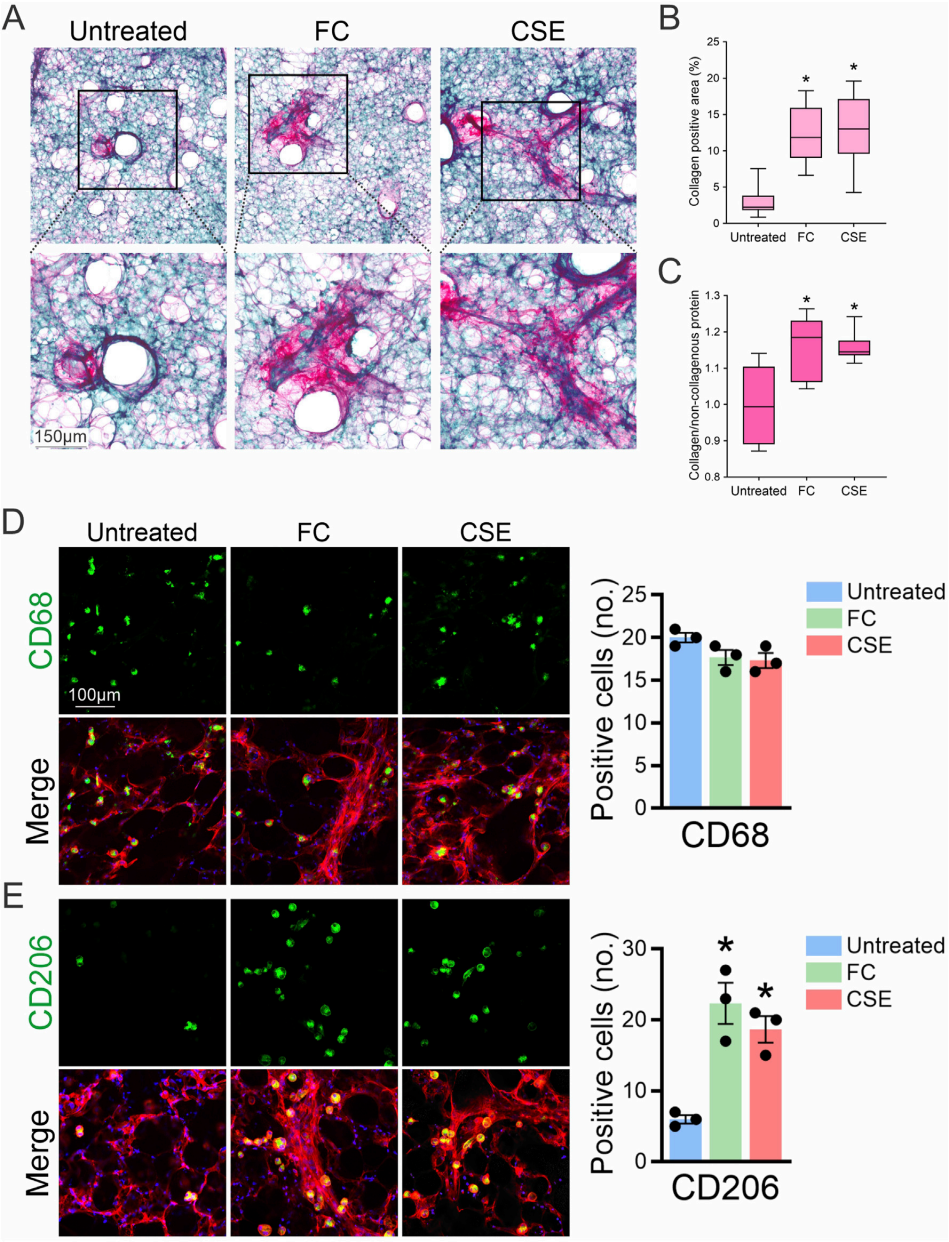
Evaluation of collagen deposition in PCLS after exposure to fibrotic inducers. **(A)** Sirius Red/Fast Green staining were used to assess collagen deposition in PCLS treated with either FC or CSE. **(B)** Quantitative analysis of Sirius Red-positive areas in lung slices (mean ± SD; n = 3; *p < 0.001 compared to the untreated group). **(C)** Collagen quantification through dye extraction from Sirius Red/Fast Green-stained sections calculated the ratio of collagen abundance relative to the untreated group (mean ± SD; n = 3; *p < 0.001). **(D, E)** PCLS from mice treated with FC or CSE were subjected to immunofluorescence staining for CD68 **(D)** and CD206 **(E)**, shown in green, and F-actin in red. DAPI (blue) marks the nucleus. Right: The number of CD68- and CD206-positive cells was quantified in 10 high-powered fields per lung slice (n = 3; *p < 0.05).

To further evaluate the extent of fibrosis in mouse PCLS exposed to FC or CSE, Sirius Red/Fast Green staining were employed to visualize collagen deposition, a key indicator of fibrotic progression. Untreated samples showed minimal collagen presence, primarily indicated by green staining. In contrast, exposure to FC significantly increased red staining, reflecting substantial collagen deposition. Similarly, PCLS exposed to CSE displayed enhanced red staining, indicating increased collagen accumulation and fibrotic changes (Figure 2A). Quantitative analysis of Sirius Red-positive areas in lung slices revealed a statistically significant increase in collagen content in both FC- and CSE-exposed groups compared to the untreated control, showing 4-fold and 4.2-fold increases, respectively, indicative of a pronounced fibrotic response (Figure 2B). Collagen quantification through dye extraction further supported these findings, showing a 1.2-fold increase in the collagen-to-non-collagenous protein ratio for FC- and CSE-exposed PCLS, significantly higher than that of the untreated control (Figure 2C). In addition, immunofluorescence staining of PCLS with CD68 confirmed that fibrosis-inducing conditions did not alter the macrophage cell number in lung tissues exposed to either FC or CSE (Figure 2D), indicating that tissue-resident macrophages (CD68^+^) remained consistent across all groups. Consistently, as the fibrotic microenvironment is known to prime macrophages toward anti-inflammatory phenotypes (Zhou et al., 2024), we observed an increase in CD206-positive cells, a marker of profibrotic M2 macrophages, in PCLS in response to both FC and CSE exposure (Figure 2E). These results underscore the distinct responses of macrophage subpopulations to fibrotic stimuli, highlighting the differential impact of pro-fibrotic agents on lung tissue. Together, these findings validate the PCLS model as an effective platform for studying fibrosis and its underlying mechanisms.

### Chemotactic response of macrophages to lung slices injured by smoke exposure

**FIGURE 3:**
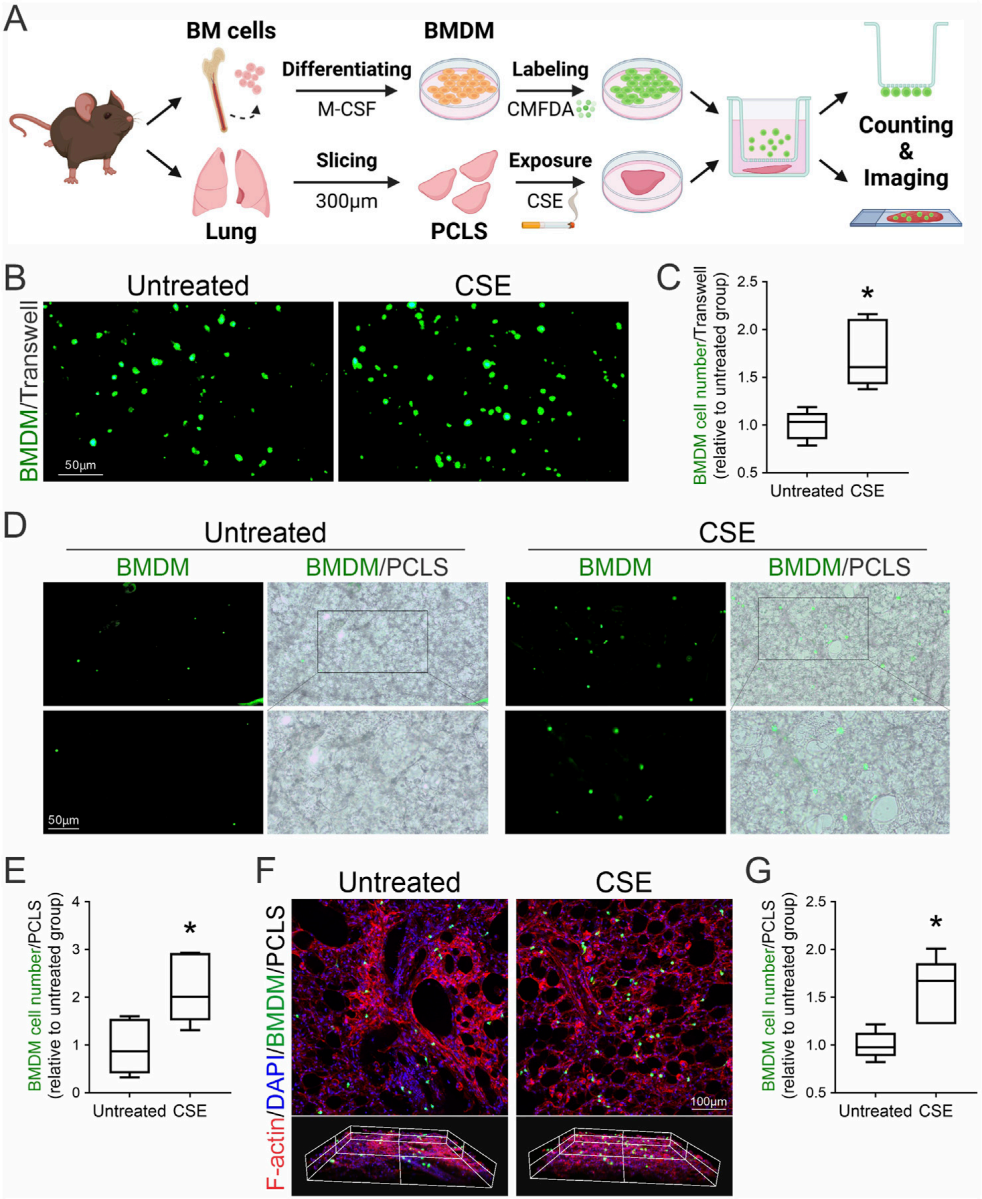
Macrophage recruitment to injured lung tissues following CSE exposure. **(A)** Schematic of chemotactic assays using autologous CMFDA-labeled BMDMs and CSE-injured PCLS. **(B)** Fluorescence microscopy was employed to visualize the migration of CMFDA-labeled BMDMs in response to PCLS treatment conditions using a transwell migration assay. **(C)** Quantitative analysis of BMDM migration was conducted by counting the number of cells that traversed the transwell membrane (mean ± SD; n = 3; *p < 0.001). **(D)** Fluorescent and bright-field images (×10 magnification) were captured to compare BMDM infiltration between untreated and CSE-treated PCLS. **(E)** Quantitative assessment of BMDM infiltration into untreated and CSE-injured PCLS as described in **(D)** (n = 3; *p < 0.05). **(F)** Immunofluorescence staining and Z-stack confocal imaging of recruited macrophages within PCLS. CMFDA-labeled BMDMs (green, infiltrated macrophages) were observed using confocal microscopy, with actin filaments stained by phalloidin (red) and nuclei counterstained with DAPI (blue). **(G)** Quantitative assessment of BMDM infiltration into untreated and CSE-injured PCLS as described in **(F)** (n = 3; *p < 0.05).

Inflammation and fibrosis involve the infiltration of monocytes into tissues, where they differentiate into macrophages through inflammation-dependent processes (Ogawa et al., 2021). To examine the chemotactic behavior of bone marrow-derived macrophages (BMDMs) in response to fibrotic stimuli, we used a transwell migration assay to mimic the inflammatory environment of lung fibrosis. Mouse BMDMs were labelled with CellTracker™ Green CMFDA and then co-cultured with injured mouse PCLS induced by exposure to CSE for 24 h. Following co-culture, the migration of BMDMs towards injured PCLS was assessed, including those that migrated across the transwell membrane and those that infiltrated into the lung tissues. Fluorescent microscopy visualized the migration of CMFDA-labeled BMDMs towards CSE-injured PCLS (Figure 3A). Under baseline conditions (untreated), minimal BMDM presence was observed, indicated by scattered fluorescent signals. In contrast, the CSE-exposed group displayed a significant increase in fluorescent signals, suggesting enhanced BMDM migration (Figure 3B). Quantitative analysis of cell migration across the transwell membrane confirmed a statistically significant increase in BMDM migration towards CSE-exposed PCLS compared to the untreated control, validating the chemotactic effect of CSE-injured lungs on macrophages (Figure 3C). Both fluorescent and bright-field images were captured to compare BMDM infiltration between untreated and CSE-exposed PCLS. In untreated samples, fluorescence imaging aligned with bright-field observations, revealing few BMDMs. In contrast, CSE-exposed samples exhibited intensified fluorescence and increased BMDM visibility in bright-field images, indicating greater infiltration (Figures 3D, E). Z-stack confocal imaging further confirmed a marked increase in BMDM recruitment into injured PCLS induced by CSE compared to healthy PCLS (Figures 3F, G). These findings confirm the pro-chemotactic influence of CSE on BMDMs within the PCLS model. The distinct migration patterns between untreated and CSE-exposed groups demonstrate the effectiveness of the PCLS model in replicating the inflammatory recruitment of immune cells seen in lung fibrosis and highlight the role of CSE as a strong stimulant of macrophage-mediated inflammation.

### Enhanced collagen deposition in PCLS co-cultured with autologous macrophages

**FIGURE 4:**
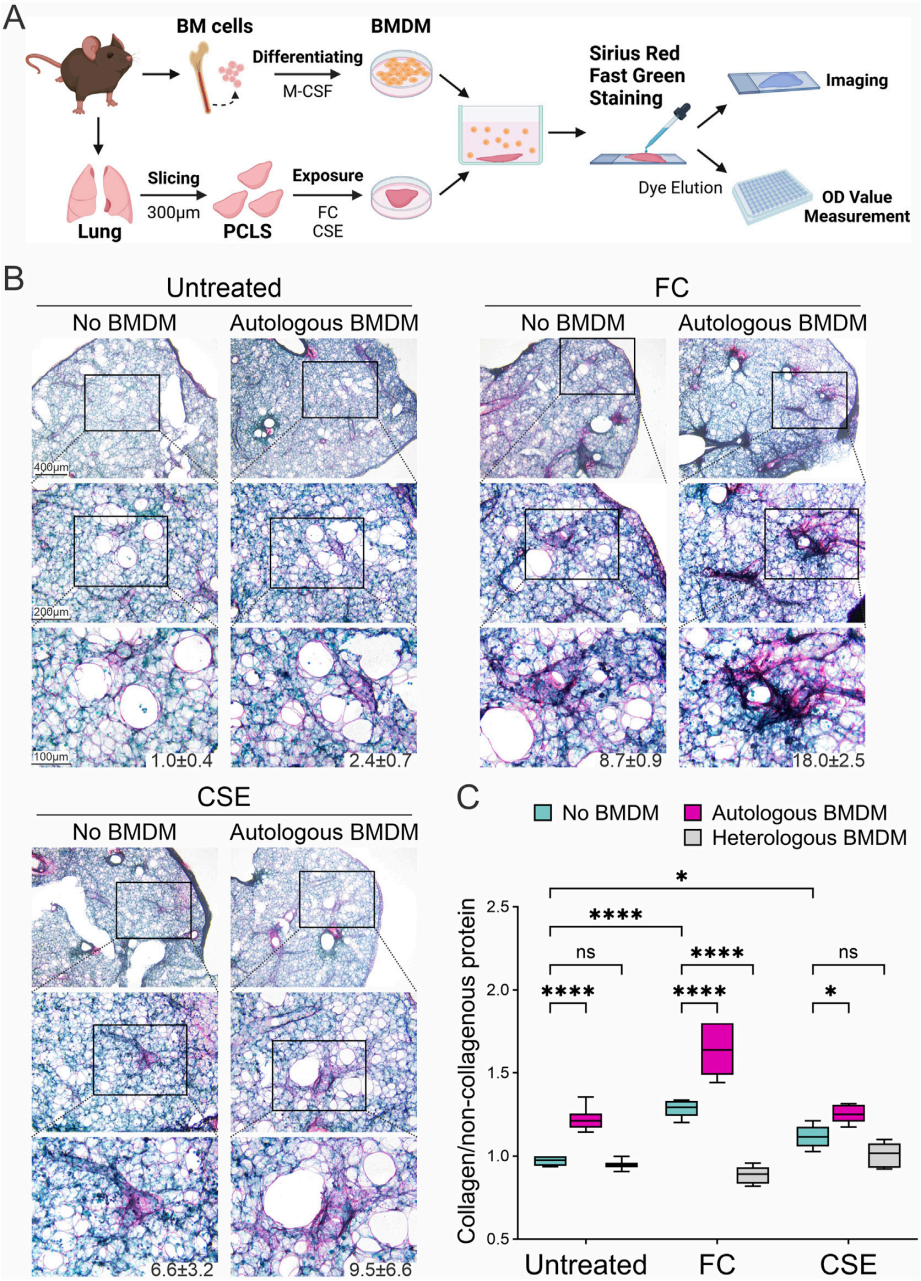
Modulation of collagen deposition through macrophage interaction in fibrotic lung tissues. **(A)** Schematic representation of direct co-cultures of BMDMs and PCLS exposed to fibrosis inducers. **(B)** Collagen deposition in PCLS was assessed histologically using Sirius Red/Fast Green staining following exposure to either FC or CSE in the absence or presence of co-cultured BMDMs. The numbers below the representative images indicate the percentage of collagen-positive area. **(C)** Quantitative analysis of collagen deposition in tissue slices was conducted by measuring the collagen protein relative to the non-collagenous protein using dye extraction from Sirius Red/Fast Green-stained sections (mean ± SD; n = 3; *p < 0.05, ****p < 0.001, ns: no significance).

To replicate the inflammation-resolution phase of fibrosis development, we established a direct co-culture of mouse PCLS with autologous BMDMs derived from the same mice. Mouse lung slices were exposed to either FC or CSE for 3 days and then directly incubated with their matching BMDMs. Collagen deposition in these lung slices was assessed histologically using Sirius Red staining after 3 days of direct co-culture (Figure 4A). Both FC and CSE exposure increased collagen deposition in PCLS even in the absence of BMDMs (No BMDM group); however, this effect was further amplified when PCLS were co-cultured with their autologous BMDMs (Autologous BMDM group). A similar pattern was observed in the CSE group, where the presence of BMDMs enhanced the fibrotic response (Figure 4B). Quantitative assessment of collagen deposition was performed using dye extraction from Sirius Red/Fast Green-stained sections to determine the ratio of collagen to non-collagenous proteins within the tissue slices. The untreated samples without BMDMs established baseline collagen expression, while the addition of autologous BMDMs significantly increased collagen deposition by 1.3-fold (Figure 4C, *p* = 0.0003). FC and CSE exposure markedly elevated the collagen-to-non-collagen protein ratio by 1.3- and 1.2-fold, respectively, an effect that was further enhanced by co-culture with autologous BMDMs (*p* = 0.0022 for FC, 1.7-fold; *p* = 0.0013 for CSE, 1.3-fold). Notably, this enhancement of collagen deposition was not observed in PCLS co-cultured with heterologous BMDMs derived from other mice (1.0-, 0.9-, and 1.1-fold, respectively), highlighting the specific impact of autologous macrophages. These findings indicate that autologous macrophages significantly enhance the fibrotic response, highlighting their role in fibrogenesis and the potential of macrophage-targeted interventions in treating fibrotic diseases.

## Discussion

This study established *ex vivo* models of pulmonary fibrosis using fibrosis inducers, enabling detailed observation of fibrotic tissue changes. Lung slices exposed to these inducers exhibited myofibroblast activation, profibrotic M2 macrophages, and increased collagen deposition. Although the role of macrophages in lung fibrosis remains debated, our indirect co-culture model revealed BMDM migration toward injured lung tissue through a transwell migration assay designed to simulate blood vessel function. In parallel, direct co-culture with autologous BMDMs significantly amplified collagen formation, underscoring their contribution to fibrogenic processes. During fibrosis induction, macrophages began polarizing to the M2 phenotype approximately 32 h post-injury. M2-activated macrophages secrete pro-fibrotic factors such as TGF-β, driving fibroblast and myofibroblast activation for collagen production (Chen et al., 2023; Chen et al., 2024; Zhou et al., 2024). Using clinically relevant fibrosis inducers such as CSE, nicotine, and FC, these models capture real-world environmental and internal factors contributing to lung fibrosis. CSE and nicotine replicate the pro-fibrotic effects of tobacco smoking, a major environmental risk factor for lung fibrosis (Baumgartner et al., 1997; Barnes et al., 2003; Bae et al., 2022), whereas FC mimics internal fibrotic processes, including inflammation, cellular proliferation, migration, and survival (Agostini and Gurrieri, 2006; Gerckens et al., 2021). These models offer a useful framework for studying the mechanisms and pathways driving fibrosis and provide insights that may inform the development of targeted therapies.

Our enhancement of the PCLS model with immune co-cultures enables a more comprehensive study of lung fibrosis in an *ex vivo* setting. By extracting lung structures from live mice, we preserved the 3D architecture and microenvironment of the lungs, allowing for the observation of cellular interactions that drive fibrotic progression. Incorporating immune cells into the PCLS system facilitated the study of immune cell recruitment, inflammation, and macrophage polarization processes. To address the limitations of traditional PCLS models, we incorporated a transwell assay to approximate blood flow, enabling the observation of immune cell recruitment to injury sites. This advancement is particularly significant given that commonly used *in vivo* models, such as bleomycin-induced pulmonary fibrosis, fail to fully replicate the irreversible nature of the disease and limit the study of all cellular players involved in fibrosis development (Liu et al., 2017; Ayilya et al., 2023; Ye et al., 2023). Our co-culture model provides a controlled platform to investigate cell-cell and cell-microenvironment interactions during inflammation and fibrosis. It allows precise manipulation of immune cell involvement and enables the addition of cytokines or therapeutic agents at defined time points, permitting real-time analysis of their effects on disease progression. By addressing the inherent limitations of *in vivo* models, this system offers a valuable tool for uncovering mechanisms underlying fibrotic diseases.

The PCLS model also aligns with the 3Rs principles (Replacement, Reduction, Refinement) by reducing animal use and minimizing variability. Multiple experimental conditions can be tested on slices from a single lung, significantly reducing the number of animals required. In our current work, we used 21 mice, nearly halving the 40 typically required in traditional *in vivo* models, demonstrating an approximately 50% reduction in animal use, with experimental reliability maintained. This approach also facilitates pre-test evaluations, ensuring efficient and ethical experimental designs while maintaining robust, reproducible results. With sustained viability for at least 2 weeks, as demonstrated in our study and supported by previous research (Alsafadi et al., 2017; Lam et al., 2023; Lehmann et al., 2024; Munyonho et al., 2024), optimizing and extending the co-culture period to 1 month could facilitate investigations into long-term responses and cumulative exposure effects, such as those caused by tobacco smoke, a critical factor in pulmonary fibrosis progression. Additionally, factors such as sex, age, and mouse strain significantly influence outcomes in murine bleomycin models and patient specimens (Redente et al., 2011; Bergeron et al., 2018; Kamio et al., 2018; Gal-Oz et al., 2019; Kato et al., 2021; Liang and Ligresti, 2023). Exploring the impact of these variables on PCLS performance represents an important avenue for future research to further refine the model and enhance its translational relevance.

We demonstrate that PCLS co-cultured with autologous macrophages exhibit enhanced collagen deposition, whereas this effect is absent in co-cultures with heterologous macrophages. This suggests that macrophages originating from the same tissue microenvironment and genetic background as the PCLS may respond more robustly to injury signals. Innate immune memory (Netea et al., 2016; Maheshwari, 2023) and preconditioning may explain the heightened responsiveness of autologous macrophages. Conversely, heterologous macrophages may lack the ability to recognize or effectively respond to tissue-specific signals (Mass et al., 2023), resulting in diminished collagen secretion. Despite the assumption that mice of the same genetic background are comparable, our results highlight the unique influence of autologous sources, demonstrating their superiority for studying immune-tissue interactions.

The involvement of macrophages in the development of lung fibrosis, as demonstrated in our study, presents them as viable targets for therapeutic strategies. The increased macrophage recruitment and collagen formation observed in Sirius Red staining confirm their significance in fibrotic repair. Identifying macrophages as key contributors to fibrotic development opens avenues for targeted therapeutic interventions (Chen et al., 2023; Hong et al., 2023; Li et al., 2023; Chen et al., 2024; Zhou et al., 2024). The indirect co-culture model, which captures macrophage migration, further underscores the importance of factors driving the recruitment process, presenting additional research targets. Given the prominent role of macrophages in lung fibrosis progression (Hong et al., 2023; Li et al., 2023), they offer a crucial target for controlling disease development. Furthermore, the variability observed in macrophage behavior within our co-cultures prompts the exploration of other immune cells and factors in fibrotic development.

While our study focuses on fibrosis aggravation and collagen deposition, future work should explore the factors released by PCLS in response to fibrotic stimuli and their interaction with macrophage-derived mediators. These factors, including cytokines and chemokines, likely play crucial roles in driving fibrotic changes and influencing macrophage recruitment and activation. Investigating the differential migration of autologous versus heterologous macrophages may uncover chemoattractants responsible for their specificity, particularly given the observed attenuation of fibrosis by heterologous macrophages in FC-induced fibrosis models. Additionally, analyzing soluble collagen levels in conditioned media could provide valuable insights into extracellular matrix (ECM) remodeling dynamics. Soluble collagen levels reflect ECM turnover (Rockey et al., 2015), influenced by macrophages through TGF-β-mediated collagen synthesis (Gibbons et al., 2011; Wynn and Ramalingam, 2012) and matrix metalloproteinase-driven degradation (Duarte et al., 2015). These investigations would enhance our understanding of macrophage-mediated fibrosis and further validate the PCLS co-culture model as a platform for studying immune cell-fibrosis interactions.

Further research is warranted to explore other environmental stressors, macrophage subtypes, and different lung diseases. Our current model, focusing on macrophage roles in fibrosis induced by CSE, nicotine, and FC, can be adapted to examine a broader spectrum of conditions. Studying the impact of various environmental stressors will elucidate similarities and differences in fibrotic pathways. Additionally, investigating different macrophage subtypes may reveal diverse interactions and roles in fibrosis progression. Since our model replicates lung architecture *ex vivo* and simulates blood flow, it can be employed to study other pulmonary diseases, potentially identifying novel targets for these conditions. Further studies are essential to validate the importance of macrophages in pulmonary fibrosis and to expand our understanding of fibrotic development and other pulmonary diseases.

In conclusion, our research enhances the current PCLS model by introducing both direct and indirect BMDM co-cultures. This innovation addresses a key limitation of the PCLS model, enabling the mimicry of immune cell responses from blood flow. We anticipate that this model will contribute significantly to the development of therapeutic treatments for pulmonary fibrosis and advance research in the field of fibrosis.

## Data Availability

The original contributions presented in the study are included in the article/Supplementary Material, further inquiries can be directed to the corresponding author.

## Abbreviations

BMDM, Bone marrow-derived macrophages; CSE, Cigarette smoke extract; DAPI, 4′,6-diamidino-2-phenylindole; DMEM/F12, Dulbecco’s Modified Eagle Medium/Nutrient Mixture F-12; PBS, Phosphate Buffered Saline; FBS, Fetal Bovine Serum; FC, Fibrosis-inducing cocktail; ITS-G, Insulin-Transferrin-Selenium; LPA, Lysophosphatidic acid; M-CSF, Macrophage colony-stimulating factor; PCLS, Precision-cut lung slices; PDGF-BB, Platelet-derived growth factor BB; TGF-β, Transforming growth factor beta; TNF-α, Tumor necrosis factor alpha.
