# Supplementary figures and images for: Autologous precision-cut lung slice co-culture models for studying macrophage-driven fibrosis
Source: Front Physiol. 2025 Jan 31;16:1526787. doi: 10.3389/fphys.2025.1526787 (PMC11825446; doi:10.3389/fphys.2025.1526787)

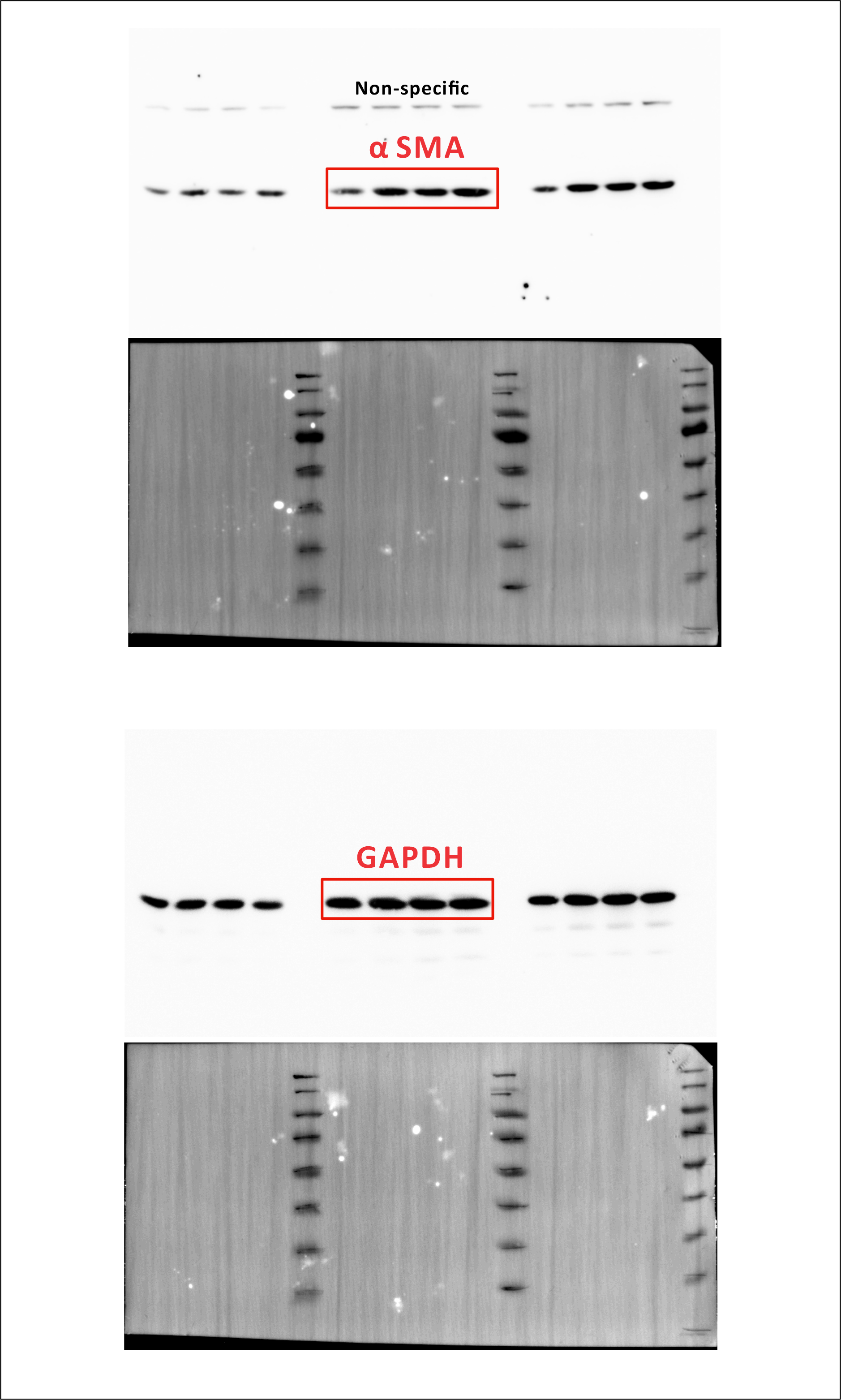

Supplement: Supplementary file 1 [file Image1.TIF]
