# Supplementary material for: Autologous precision-cut lung slice co-culture models for studying macrophage-driven fibrosis
Source: Front Physiol. 2025 Jan 31;16:1526787. doi: 10.3389/fphys.2025.1526787 (PMC11825446; doi:10.3389/fphys.2025.1526787)
Supplement: Supplementary file 2 [file DataSheet1.PDF]

Online Data Supplement for

Autologous precision-cut lung slice co-culture models for studying macrophage-driven fibrosis

So-Yi Chang, Wen-Hsin Chang, David C. Yang, Qi-Sheng Hong,
Ssu-Wei Hsu, Reen Wu, Ching-Hsien Chen*

Supplementary Materials and Methods

Reagents and antibodies

Dulbecco's Modified Eagle's Medium/F12 (DMEM/F12), RPMI medium, fetal bovine serum (FBS), Insulin-Transferrin-Selenium (ITS-G), penicillin-streptomycin, and Alexa Fluor 568 Phalloidin were obtained from Gibco, ThermoFisher Scientific (Waltham, MA, USA). Lysophosphatidic Acid (LPA) was obtained from Santa Cruz Biotechnology Inc (Santa Cruz, CA, USA) Anti-GAPDH, anti-CD68, anti-CD206 antibodies were acquired from Cell Signaling Technology (Danvers, MA, USA), and anti-smooth muscle actin (α-SMA) antibodies were sourced from American Research Products, Inc. (ARP, Waltham, MA, USA). Recombinant mouse TGF-β, TNF-α, PDGF-BB, and M-CSF were procured from BioLegend (San Diego, CA, USA).

Isolation of mouse bone marrow-derived macrophages (BMDMs)

Femurs and tibiae from 8-week-old male mice were used to flush out bone marrow using RPMI medium with a 25-gauge syringe. After filtration and erythrocyte removal, the bone marrow cells were cultured in RPMI medium supplemented with 40 ng/mL M-CSF for three days. The medium was then replaced with a mixture of 20% L929 cell-conditioned medium and 20 ng/mL M-CSF for an additional four days. After seven days of differentiation, BMDMs were collected for subsequent experiments.

Ex vivo co-culture system of bone marrow-derived macrophages and PCLS

This *ex vivo* model, designed to investigate acute inflammation recruitment, utilized a transwell system (5 µm pore size, Costar, Cambridge, MA, USA). The transwell membrane thickness is proprietary to the manufacturer. A 24-hour incubation period was chosen to allow macrophage activation and migration while minimizing proliferation, based on macrophage doubling times of 20-30 hours (Chitu et al., 2011) and chemotaxis studies supporting migration within 2-24 hours (Arts and Netea, 2016). Injured PCLS in the bottom chamber mimic a distal injured lung. Initially, 8×10^5 CMFDA fluorescent dye-labeled BMDMs (Thermo Fisher Scientific, Waltham, MA, USA) were placed onto the transwell filters, while PCLS treated with CSE were situated in the lower well. After a 24-hour incubation period, cell counts were performed on the lower surface of the filter using a fluorescent microscope at 20× magnification. The PCLS located in the lower chamber, infiltrated by green-fluorescent BMDMs, were fixed in 4% paraformaldehyde for 25 minutes and subsequently imaged with a fluorescent microscope at 10× magnification. To evaluate the role of infiltrated immune cells during the resolution phase, well-prepared PCLS were initially exposed to CSE or FC for three days, after which 2×10^5 BMDM cells were added. The PCLS were then harvested following an additional three days of culture.

Sirius Red/Fast Green collagen staining

For collagen detection in PCLS, we utilized a modified protocol from the Sirius Red/Fast Green collagen staining kit (Chondrex, WA, USA). PCLS treated with FC and CSE for six days were washed with PBS and then fixed in 4% paraformaldehyde for 20 minutes. Following another PBS wash, the fixed PCLS were incubated with the Dye Solution at room temperature for 30 minutes, and subsequently rinsed with distilled water until the rinse water was clear. For dye extraction, 1 mL of Dye Extraction Buffer was added to each sample and gently mixed by pipetting until the dye was fully eluted. The eluted dye solution was then collected, and optical density (OD) values were measured at 540 nm and 605 nm using a spectrophotometer. In an alternative

processing step, the stained slices were sequentially dehydrated with 85%, 95%, and 100% ethanol, mounted in toluene, and examined under a microscope. Histological quantification was conducted in multiple random areas per section and reviewed by an independent individual blinded to the sample identities.

Immunofluorescence and Immunoblotting

PCLS treated with nicotine, CSE, and FC for six days were washed with PBS and then subjected to either immunofluorescence or immunoblotting. For immunofluorescence, PCLS were fixed in 4% paraformaldehyde for 20 minutes and washed again. The fixed PCLS were incubated overnight at 4°C with primary antibodies against α-SMA, CD68, and CD206. Subsequently, the sections were incubated with Alexa Fluor 568 Phalloidin (F-actin) and Alexa 488-labeled secondary antibodies for 1 hour at room temperature in the dark. Nuclei were counterstained with DAPI, and the slides were mounted in a mounting medium. Immunofluorescence was performed using the FluoView FV3000 spectral confocal system (Olympus, Center Valley, PA, USA). For immunoblotting, total lung slice lysates were prepared using T-PER™ Tissue Protein Extraction Reagent (Thermo Fisher Scientific, Waltham, MA, USA). Proteins were separated via SDS-PAGE, transferred to PVDF membranes, probed with appropriate antibodies, and visualized using enhanced chemiluminescence. Immunoblotting images were quantified using ImageJ software (National Institutes of Health, Bethesda, MD, USA).
